# Functional Limitations of Plasmacytoid Dendritic Cells Limit Type I Interferon, T Cell Responses and Virus Control in Early Life

**DOI:** 10.1371/journal.pone.0085302

**Published:** 2013-12-23

**Authors:** Elodie Belnoue, Paola Fontannaz, Anne-Françoise Rochat, Chantal Tougne, Andreas Bergthaler, Paul-Henri Lambert, Daniel D. Pinschewer, Claire-Anne Siegrist

**Affiliations:** World Health Organization Collaborating Center for Vaccinology and Neonatal Immunology, Departments of Pathology-Immunology and Pediatrics, University of Geneva, Geneva, Switzerland; Kantonal Hospital St. Gallen, Switzerland

## Abstract

Infant mortality from viral infection remains a major global health concern: viruses causing acute infections in immunologically mature hosts often follow a more severe course in early life, with prolonged or persistent viral replication. Similarly, the WE strain of lymphocytic choriomeningitis virus (LCMV-WE) causes acute self-limiting infection in adult mice but follows a protracted course in infant animals, in which LCMV-specific CD8^+^ T cells fail to expand and control infection. By disrupting type I IFNs signaling in adult mice or providing IFN-α supplementation to infant mice, we show here that the impaired early life T cell responses and viral control result from limited early type I IFN responses. We postulated that plasmacytoid dendritic cells (pDC), which have been identified as one major source of immediate-early IFN-I, may not exert adult-like function *in vivo* in the early life microenvironment. We tested this hypothesis by studying pDC functions *in vivo* during LCMV infection and identified a coordinated downregulation of infant pDC maturation, activation and function: despite an adult-like *in vitro* activation capacity of infant pDCs, the expression of the E2-2 pDC master regulator (and of critical downstream antiviral genes such as MyD88, TLR7/TLR9, NF-κB, IRF7 and IRF8) is downregulated *in vivo* at baseline and during LCMV infection. A similar pattern was observed in response to ssRNA polyU, a model ligand of the TLR7 viral sensor. This suggests that the limited T cell-mediated defense against early life viral infections is largely attributable to / regulated by infant pDC responses and provides incentives for novel strategies to supplement or stimulate immediate-early IFN-α responses**.**

## Introduction

The early life period is characterized by an increased vulnerability to viral pathogens such as influenza, respiratory syncytial virus, HSV-1 or human cytomegalovirus. Indeed, these viral infections run a more severe and prolonged course in infants [1,2] in whom CD8^+^ T cells only appear at time of recovery and convalescence [3,4]. This has long been thought to result from the incapacity of the neonatal immune system to raise protective T cell responses as a result of its programming for neonatal tolerance induction [5]. However, recent work has suggested that innate immune responses are distinctly regulated in early and adult life, the vulnerability to pathogens resulting from the need to avoid eliciting harmful inflammatory, alloreactive and autoimmune responses at a vulnerable period of life [6,7]. Identifying the molecular and cellular bases of the immune regulation of early life immune responses, and strategies to safely improve defense against pathogens, is thus of considerable interest. 

To identify the mechanisms underlying the enhanced severity of viral infections in early life, we investigated lymphocytic choriomeningitis virus (LCMV) infection in its natural murine host. LCMV is strictly non-cytolytic and runs an acute, chronic or persistent course of infection dependent upon the host immune responses. In immunocompetent adult mice, low doses of the LCMV-WE strain are rapidly cleared. Conditions where T cell responses are impaired (including in utero or within 24h of birth) avoid immunopathological reactions but result in chronic asymptomatic infections [8]. In 2-week-old infant mice, whose stage of immune maturation is no more neonatal nor yet adult-like, LCMV-WE follows a protracted course characterized by several weeks of viral replication eventually terminated by the onset of neutralizing antibodies [9]. We previously documented that the protracted LCMV infection of 2-week-old mice correlates with their failure to mount the adult-like antiviral CD8^+^ T cell responses required for rapid LCMV clearance [9]. 

Plasmacytoid dendritic cells (pDCs) play pleiotropic roles in viral infections. By producing type I IFNs (IFNs α and β, IFN-I) within hours of TLR-mediated signaling, they promote the apoptosis of infected cells, activate NK cells and protect conventional dendritic cells (cDCs) and macrophages - thus rapidly controlling viral loads [10-13]. IFN-I promote the expansion of CD4^+^ T cells through direct and indirect (APC-mediated) effects [14,15] and participate in the induction CD8^+^ T cell responses [16]. They may substitute for CD4^+^ T cell help [17,18] by allowing CD8^+^ T cells to escape from TRAIL-mediated apoptosis [19,20], thus supporting their expansion. We thus postulated that pDCs may not exert adult-like function *in vivo* in the early life microenvironment despite their *in vitro* activation [21-23]. We tested this hypothesis by studying pDC functions *in vivo* during LCMV infection. We demonstrate here that pDC activation is indeed downregulated in response to LCMV infection and model TLR7 ligands. This prevents the immediate-early burst of IFN-I required for early viral control and subsequent CD8^+^ T cell expansion, thereby setting the stage for protracted courses of viral infections. 

## Materials and Methods

### Ethics statement

Manipulations of mice were carried out according to Swiss and European guidelines and all experiments were approved by the Geneva Veterinary Office.

### Mice

BALB/c mice were purchased from Charles River (L’Arbresle, France) and kept under specific pathogen free conditions. IFN-I receptor deficient (IFNAR^-/-^) mice [24] in BALB/c background were kindly given by Rainer Zawatzky (Heidelberg, Germany). Breeding cages were checked daily and the day of birth was recorded as the day the litter was found. Pups were kept with mothers until weaning at the age of 4 weeks. Adult mice were used at 8 to 12 weeks of age. 

### LCMV, infection and peptides

Infection was performed by i.v. injection of 100pfu of LCMV-WE. In one experiment, 10pfu were injected. Virus titers of infected spleens were determined in a virus plaque assay as previously described [9]. Virus titers are expressed as pfu per gram of spleen. Peptides carrying the immunodominant epitope p118-126 (LCMVnp118-126) or the CD4 epitope p6-20 (LCMVnp6-20), both from the LCMV strain WE nucleoprotein were synthesized by PolyPeptide Laboratories (Strasbourg, France).

### FACS staining

Single cell suspensions were obtained from spleens of infected animals. Specific staining of TCR was performed by using dimeric MHC class I-peptide complexes according to the manufacturer’s instructions (BD Pharmingen). FACS staining was performed with following antibodies: anti-CD11c (clone HL3, BD Pharmingen), anti-B220 (clone RA3-6B2, BD Pharmingen), anti-PDCA-1 (clone JF05-1C2.4.1, Miltenyi Biotec GmbH, Bergisch Gladbach, Germany), anti-Siglec-H (clone 551), anti-CD40 (clone FGK45), anti-CD80 (clone 16-10A1, BD Pharmingen), anti-CD86 (clone GL-1), anti-CD8 (clone 53-6.7, BD Pharmingen), anti-CD4 (clone RM4-5, BD Pharmingen), anti-CD44 (clone IM7, BD Pharmingen), anti-IFN-γ (clone XMG-1, BD Pharmingen), anti-IL2 (JES6-5H4, BD Pharmingen), anti-TNF-α (clone MP6-XT22, BD Pharmingen). Cells were analyzed using the FACSAria (BD Biosciences) or the Gallios (Beckman Coulter) and FlowJo Software (Tree Star, Ashland, OR). Fold increases were calculated as follows: the geometric mean fluorescence intensity of each marker was divided by the geometric mean fluorescence intensity of the isotype control, and fold increases obtained by dividing day 1 by day 0 values. For intracellular analysis, 2x10^6^ of splenocytes were cultured with control medium, LCMVnp118-126 (10μg/ml) or LCMVnp6-20 (20μg/ml) peptides for 7h at 37°C in presence of anti-CD28 (clone 37.51, BD Pharmingen) and anti-CD49d (clone 9C10, BD Pharmingen) for CD4^+^ T cell responses. Brefeldin A (Sigma-Aldrich, Buchs, Switzerland) and monensin were added for the last 6 hours. Following staining for surface antigens, cells were stained for cytokines using the cytofix/cytoperm kit (BD Pharmingen). 

### IFN-α

IFN-α content was measured in the sera by capture ELISA kit (PBL Biomedical Laboratories, NJ) following manufacturer’s instructions. Values were expressed by reference to a standard curve constructed by assaying serial dilution of recombinant IFN-α. Ten thousands units of recombinant mouse IFN-α (Merck KGaA, Darmstadt, Germany) was injected 4h, 8h and 12h after infection. In another set of experiments, an additional single injection of 1μg of pegylated human IFN-α 2a (Pegasys, Roche Pharma, Reinach, Switzerland) was added at 6h.

### Cell purification

Untouched pDCs were isolated from spleens by using Plasmacytoid Dendritic Cell isolation kit II (Miltenyi Biotec GmbH) and following the manufacturer’s instructions generating an enriched population containing over 83% pDCs. For pDC sorting, spleens were mechanically digested, ACK-treated and CD19^+^ B cells and CD3^+^ T cells depleted using MACS beads (Miltenyi Biotec GmbH). Remaining cells were stained using B220, PDCA-1, CD11c and Siglec-H markers. pDCs were sorted using Moflo Astrios (Beckman Coulter) as CD11c^int^B220^+^PDCA-1^+^Siglec-H^+^ cells with a purity ≥ 95%. Splenic conventional DCs (after DNAse/liberase digestion) were isolated by the use of anti-CD11c MACS beads (Miltenyi Biotec GmbH), reaching purities of over 90%. 

### Cytospin and staining

Isolated untouched pDCs were cytospined and fixed in cold methanol. IRF-7 staining was performed using rabbit anti-mouse IRF-7 Ab (clone EPR4718, GeneTex Inc., Irvine, CA), Alexa 488-conjugated anti-rabbit IgG (Molecular Probes, Eugene, OR) and transcription factor buffer set (eBioscience, San Diego, CA). Nuclei were counterstained with DAPI. 

### *In vitro* and *in vivo* responses to PolyU

Isolated untouched pDCs were stimulated o.n. with different concentrations of PolyU ssRNA / polyethylenimine or polyethylenimine (Sigma-Aldrich) alone. Infant and adult mice were injected i.v. with weight-adjusted PolyU (6 and 15μg, respectively) complexed with DOTAP (20 and 50μl, respectively; Roche Diagnostics, Mannheim, Germany). 

### Real-Time PCR

Total cellular RNA from total spleens, cDCs or flow-sorted pDCs was isolated by RNeasy mini kit or micro kit respectively. cDNA was synthesized from 0.5μg of total RNA using a mix of random hexamers–oligo d(T) primers and PrimerScript reverse transcriptase enzyme (Takara bio inc. Kit). For isolated pDCs, pre-amplification was performed with TaqMan® PreAmp Master Mix following supplier's instructions (Applied Biosystems). PCR reactions contained diluted cDNA, 2 x Power SYBR Green Master Mix (Applied Biosystems), 300nM of forward and reverse primers and were performed on a SDS 7900 HT instrument (Applied Biosystems). Each reaction was performed in three replicates, with *ActinG, EEf1, gapdh* and *GusB* as internal controls genes for data normalisation. Raw Ct values obtained with SDS 2.2 (Applied Biosystems) were imported in Excel and normalisation factor and fold changes were calculated using the GeNorm method. The efficiency of the primer design was tested with serial dilutions of cDNA and found >90% for all primer pairs. The following primers (Invitrogen) were used: IFN-α-forward 5’-CCTGAGAGAGAAGAAACACAGCC-3’ and IFN-α-reverse 5’- TTCTGCTCTGACCACCTCCC-3’; IRF-7-forward 5’-GGGAGGCCCAAGGAGAAG-3’ and IRF-7-reverse 5’-CATACCCATGGCTCCAGCTT-3’; MyD88-forward 5’-CCTTGATGACCCCCTAGGACA-3’ and MyD88-reverse 5’-CAGATAAAGGCATCGAAAAGTTCC-3’; TLR7-forward 5’-GATCGTGGACTGCACAGACAA-3’ and TLR7-reverse 5’-GGAATGCCCTCAGGGATTTC-3’; TLR9-forward 5’-GGCCCATTGTGATGAACCTG-3’ and TLR9-reverse 5’-GCAGAGGGTTGCTTCTCACG-3’; NF-kappaB-reverse 5’-CTGGACATGGCTGCCAACT-3’ and NF-kappaB-reverse 5’-CGGTTTCCCATTTAGTATGTCAAA-3’; IL-6-forward 5’-CTATGAAGTTCCTCTCTGCAAGAGACT-3’ and IL-6-reverse 5’-GGGAAGGCCGTGGTTGTC-3’; TNF-alpha-forward 5’- GACCCTCACACTCAGATCATCTTCT-3’ and TNF-alpha-reverse 5’-CCACTTGGTGGTTTGCTACGA-3’; E2-2-forward 5’-CCTGACTTGAACCCACCCC-3’ and E2-2-reverse 5’-GAGGCCTGGTGGCATCC-3’; IRF-8-forward 5’-CAGGTCTTTGACACCAACCAGTT-3’ and IRF-8-reverse 5’-GGCGTAGAATTGCTGCAGC-3’. Four house-keeping genes (Actin-gamma, EEF1A1, Gapdh, GusB), observed as similarly expressed in infant and adult samples (not shown), were used for gene normalization between samples.

### Statistical Analysis

Statistical differences between two groups were analyzed by Mann-Whitney U test, differences between multiple groups were analyzed by one-way ANOVA followed by the Tukey multiple comparison test and differences with probability values < 0.05 were considered significant.

## Results

### Delayed production of IFN-I and impaired viral control in 2-week-old mice

**Figure 1:**
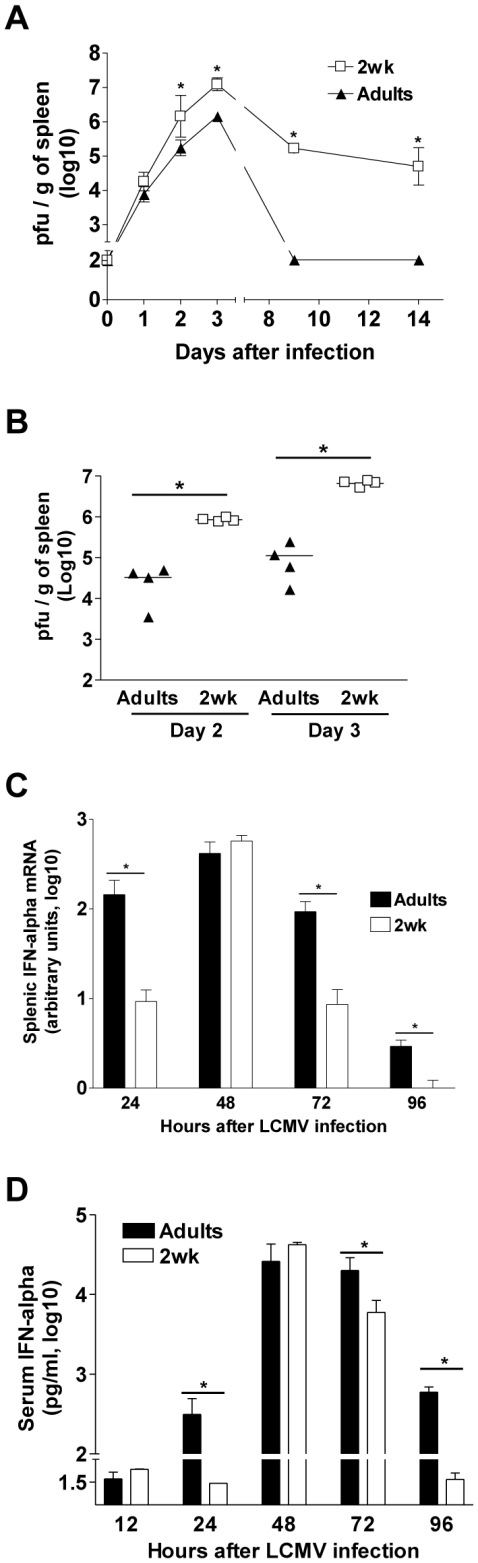
Impaired early viral control is associated to a delayed/shorter IFN-α burst in infant mice. (A) Spleen viral titers at different time points after infection of 2-week-old and adult mice infected i.v. with 100 pfu of LCMV-WE. One of two representative experiments is shown. *, *p*<0.05 versus adult mice. (B) Spleen viral titers at day 2 and 3 after infection of 2-week-old and adult mice infected i.v. with a reduced dose (10 pfu) of LCMV-WE. One of two representative experiments is shown. *, *p*<0.05. (C) Relative IFN-α mRNA expression quantified in the spleen at different time points after infection. One of two representative experiments is shown. *, *p*<0.05. (D) IFN-α titers quantified by ELISA in the serum at different time points after infection. One of three representative experiments is shown. *, *p*<0.05.

To define the contribution of innate responses to the fate of viruses which run a protracted course in early life, we infected 2-week-old BALB/c mice with LCMV-WE [9]. Assessing the pattern of early viral control (day 2-3), we observed markedly higher (at least 1 log_10_) splenic LCMV-WE viral titers than in adult mice (Figure 1 A). A similar pattern was observed when the infectious dose was reduced from 100 to 10 pfu (Figure 1 B). IFN-I rapidly restrict viral replication and play a critical role in controlling LCMV infection [24]. In adult mice, IFN-α was already detected in the spleen and serum at 24h, peaked at 48h and remained detectable up to 72h-96h after infection (Figure 1 C and D) as described [25]. In 2-week-old mice, IFN-α remained significantly lower at 24h, transiently reached adult levels at 48h but returned to baseline levels earlier than in adult mice (Figure 1 C and D). Thus, an immediate-early burst of IFN-α occurred before the peak of LCMV-WE in adults but was delayed and shorter in 2-week-old mice.

### IFNAR^-/-^ mice recapitulate defective T cell responses and virus control of infant animals

**Figure 2:**
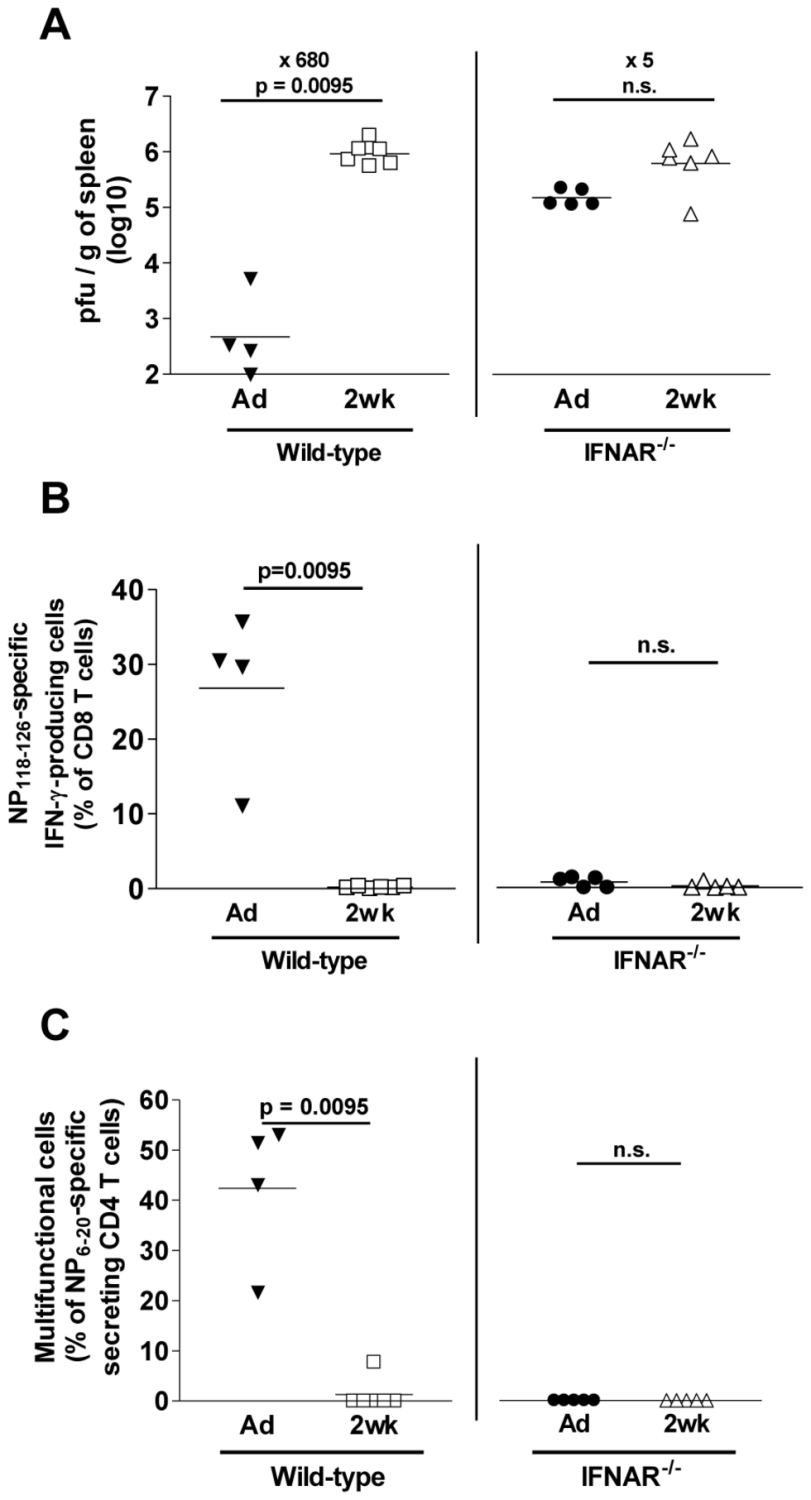
Similarly impaired viral control and T cell responses in infant WT as in adult/infant IFNAR^-/-^ mice. 2-week-old and adult WT or IFNAR^-/-^ mice were infected i.v. with 100 pfu of LCMV-WE and assessed 10 days later. (A) Spleen viral titers. One of two representative experiments is shown. The fold difference in viral titers between adult and infant WT (x 680) or IFNAR-/- (x 5) mice is indicated. (B) Percentage of LCMVnp118-126-specific IFN-γ producing CD8^+^ T cells. Percentages obtained in medium controls were subtracted. One of two representative experiments is shown. (C) % of LCMVnp6-20-specific IFN-γ, IL-2 and TNF-α secreting CD44^+^CD4^+^ T cells. Percentages obtained in medium controls were subtracted. One of two representative experiments is shown.

To define whether the limitation of early life IFN-I responses were sufficient to explain alone the early life patterns of CD4/CD8 T cell responses and viral control, we compared viral titers and T cell responses in LCMV-infected 2-week-old vs. adult wild-type (WT) or IFN-I receptor-deficient (IFNAR^-/-^) mice. On day 10, splenic LCMV titers had already declined to very low levels in adult WT mice. In contrast, they persisted at similarly high titers in adult and infant IFNAR^-/-^ as in WT infant mice. Slightly lower but statistically similar titers were observed in adult and infant IFNAR^-/-^ mice (Figure 2 A), suggesting a main role of IFN-I signaling in the shaping of the infant viral pattern. In accordance with the role of IFN-I on T cell clonal expansion [26], this viral pattern was mirrored by the frequencies of IFN-γ-producing LCMV-specific CD8^+^ T cells which were similarly reduced in 2-week-old WT, 2-week-old IFNAR^-/-^ and adult IFNAR^-/-^ mice on day 10 (Figure 2 B). CD4^+^ T cell responses showed a comparable deficit (Figure 2 C). Thus, the limited early life IFN-α responses were associated to a similar pattern of impaired viral control / limited T cell responses as in adult (and infant) IFNAR^-/-^ mice.

### Limited pDC response to LCMV in infant mice

**Figure 3:**
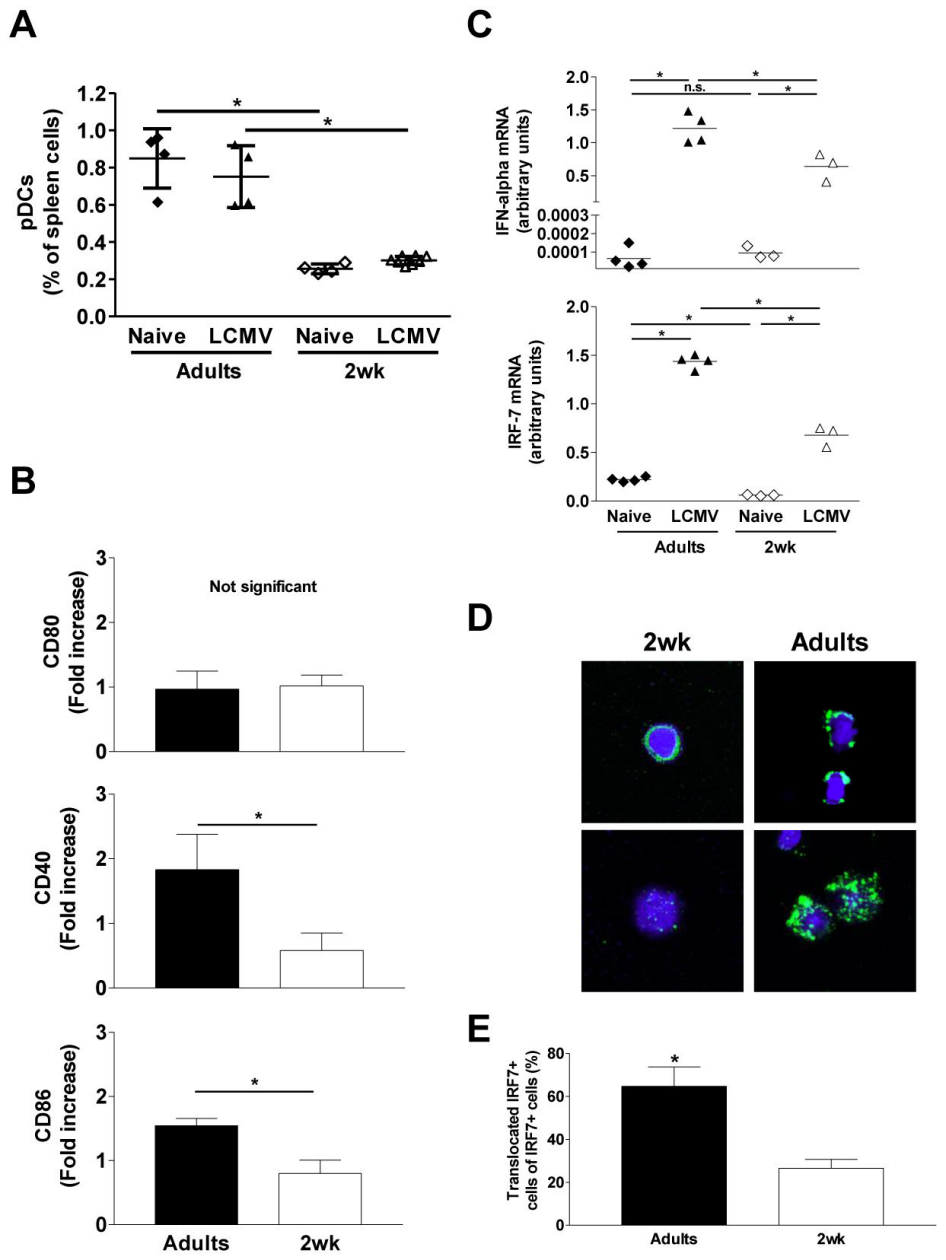
Limited activation and function of pDCs in LCMV-infected 2-week-old mice. (A) Percentages of infant and adult splenic CD11c^int^Siglec-H^+^B220^+^PDCA-1^+^ pDCs determined by FACS staining before (naive) and 1 day after LCMV-WE infection (LCMV). One of three representative experiments is shown. *, *p*<0.05. (B) Fold increase of CD80, CD40 and CD86 expression by infant and adult splenic pDCs, determined by FACS staining between day 0 and day 1 after LCMV-WE infection. *, *p*<0.05. n = 6 to 8 mice per group. One of two representative experiments is shown. (C) Expression of IFN-α, and IRF-7 mRNAs in sorted pDCs from infant or adult spleens before (naive) and 1 day after LCMV-WE infection (LCMV). *, *p*<0.05. n = 4 pools of 2 adult mice or 3 pools of 6 to 8 infant mice. One of two representative experiments is shown. (D) Pictures of isolated pDCs from infected infant and adult mice after cytospin, methanol fixation and IRF-7 (green) and DAPI (blue) staining (EC Plan Neofluar 40x 1.3 Oil DIC objective, Zeiss LSM510 Meta confocal microscope). Upper and lower panels showing non-translocated and translocated nuclear IRF-7, respectively. (E) Percentage of IRF-7^+^ pDCs displaying IRF-7 translocation into the nucleus. *, p<0.05. More than 1000 IRF7^+^ pDCs (from at least 4 different individual adult or 4 different pools of 6 to 8 infant mice) were counted. One of two representative experiments is shown.

pDCs have been identified as one major source of immediate-early IFN-I in LCMV-infected adult mice [25,27-29]. We thus assessed splenic CD11c^int^Siglec-H^+^B220^+^PDCA-1^+^ pDCs by FACS at day 0 and 1 after LCMV-WE infection. At baseline, the percentages (Figure 3 A) and numbers of pDCs were significantly higher in adults (712x10^3^ ± 110) than in infant (90x10^3^ ± 27) mice. As previously described [30], LCMV infection did not increase the percentage (Figure 3 A) and the number of pDCs (data not shown). An upregulation of CD40 and CD86 (but not CD80) expression reflected the LCMV-induced activation of adult but not infant pDCs (Figure 3 B). IFN-α mRNA expression increased from very low baseline levels to significantly higher levels in splenic pDCs of LCMV-infected adult mice, as described [31]. Despite a strong increase of IFN-α mRNA expression in infant pDCs following LCMV infection, its expression remained significantly lower than in pDCs from LCMV-infected adults (more than 2-fold difference, Figure 3 C, upper panel) – explaining the reduced level of immediate-early burst of serum IFN-α in early life (Figure 1 C). 

IFN-I production requires the expression, phosphorylation and nuclear translocation of IFN Regulatory Factor (IRF-) 7, the master regulator of IFN-I [32]. LCMV infection indeed significantly increased the expression of IRF-7 in both adult and infant pDCs but constitutive and post-infection levels remained significantly lower in infant pDCs (Figure 3 C, lower panel). Furthermore, IRF-7 was less efficiently translocated into the nucleus of infant pDCs (Figure 3 D and E). Thus, the lack of an immediate-early burst of IFN-α in LCMV-infected infant mice correlated with limited pDC activation, IRF-7 expression and nuclear translocation. 

### The limited activation of infant pDCs correlates with low levels of the master transcription factor E2-2

**Figure 4:**
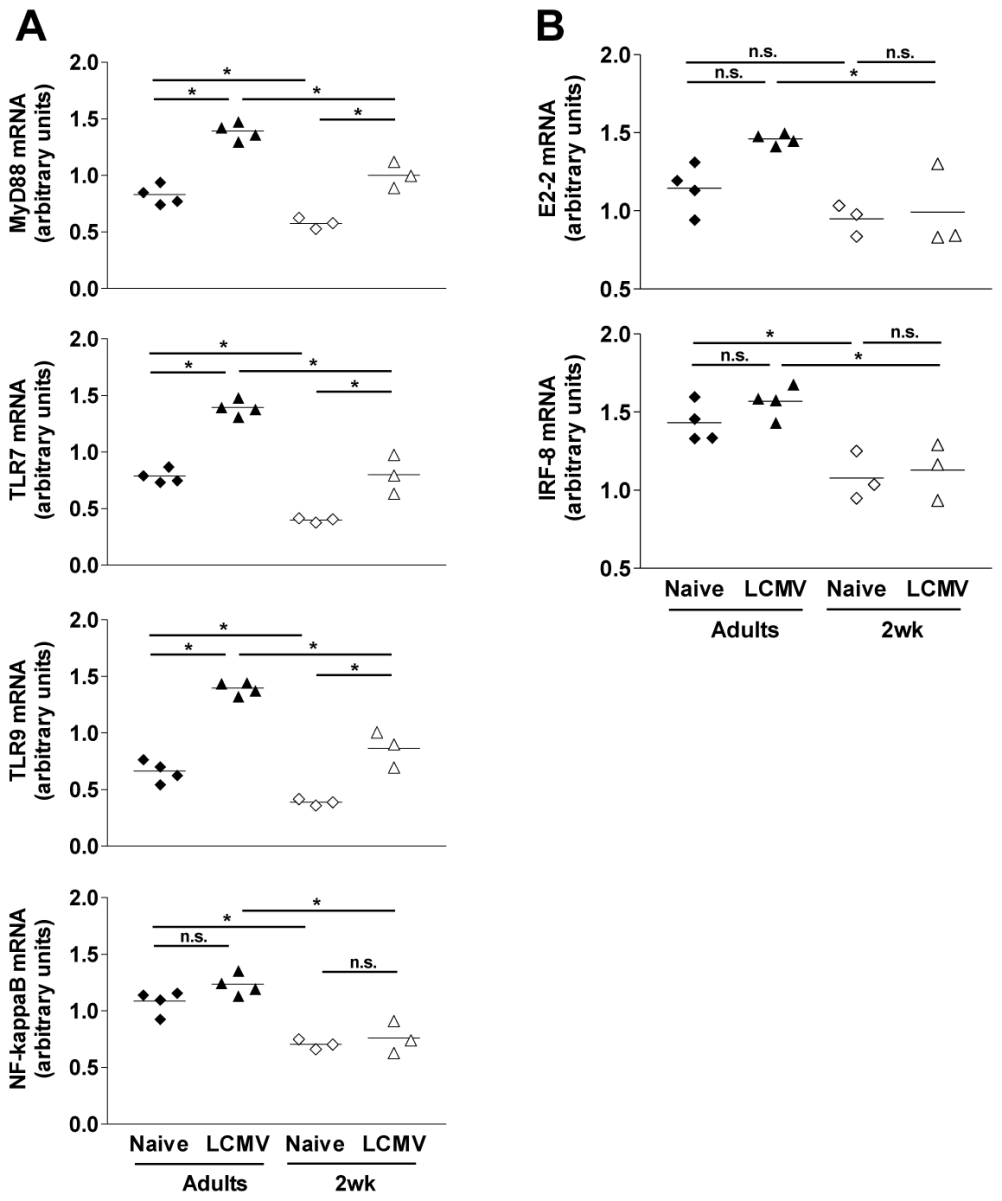
Downregulation of critical antiviral genes in infant pDCs. mRNA expression of MyD88, TLR7, TLR9 and NF-κB (A) or E2-2 and IRF-8 (B) in sorted pDCs from spleens of infant or adult mice before (naïve) and 1 day after LCMV infection (LCMV). *, *p*<0.05. n = 4 pools of 2 adult mice or 3 pools of 6 to 8 infant mice. One of two representative experiments is shown.

To understand how both IRF-7 expression and translocation were limited in infant pDCs, we assessed the expression of three critical pDC molecules: MyD88, a docking site for proteins implicated in IRF-7 phosphorylation, and TLR7 and TLR9 which recognize viral nucleic acids and trigger MyD88 recruitment [33]. Infant pDCs displayed lower constitutive levels of each of these 3 genes compared to adult pDCs (Figure 4 A). Conversely, Siglec-H levels were comparable (data not shown) indicating similar pDC purity. The expression of MyD88, TLR9 and TLR7 (recently demonstrated as mediating the pDC response to LCMV [31]) increased during LCMV-WE infection but only enabled infant pDCs to reach the levels observed at baseline in adult pDCs (Figure 4 A). As MyD88, TLR7 and TLR9 are critical for NF-κB activation, we assessed its expression and that of two NF-κB-regulated cytokines, IL-6 and TNF-α. Again, both the constitutive and inducible expression of NF-κB (Figure 4 A, lower panel), IL-6 and TNF-α (data not shown) remained at significantly lower levels in infant than adult pDCs. 

To define the pDC specificity of transcriptional control of these genes in early life, the expression of IFN-α IRF-7, TLR9, TLR7, NF-κB and MyD88 mRNA was also quantitated in infant and adult cDCs before and 3 days after LCMV infection. The upregulation of IFN-α transcription was also lower in infant than in adult cDCs (fold increase 6.9 ± 1.1 and 18.5 ± 2.8 respectively, *p* = 0.0079) but much less impressive than in infant and adult pDCs (fold increases: 5810 ± 1525 and 19220 ± 3640 respectively; *p* = 0.0038), indicating that the early life limitation of IFN-α expression may involve other cell types. These blunted responses may contribute to the lower levels of IFN-α observed at later time points after LCMV infection (Figure 1 C-D). Whether this significantly impacts viral control and T cell responses is unclear given the lower IFN-α responses by cDCs than pDCs and the critical role of the immediate-early pDC-derived IFN-α production in infection control [25]. Importantly, infant cDCs expressed adult levels of IRF-7, TLR9, TLR7, NF-κB and MyD88 mRNAs (data not shown), confirming the pDC specificity of the gene expression control. 

The unexpected observation of the limited expression of several molecules critical for pDC activation/function indicated that numerous pathways were specifically down-regulated in infant pDCs. The E protein E2-2 directly activates multiple transcription factors involved in pDC development and function [34,35]. Its quantification identified a significantly reduced expression of E2-2 in early life pDCs during LCMV infection (Figure 4 B). The IRF-8 transcription factor (which is directly activated by E2-2 in pDCs) was only weakly expressed in infant compared to adult pDCs (Figure 4 B). Thus, the constitutive expression and *in vivo* activation of critical pDC genes controlled by E2-2 were specifically downregulated in early life pDCs. This likely prevents the generation of an immediate-early burst of IFN-I, impairs the initial control of LCMV and limits T cell expansion, resulting into a protracted infection. 

### Infant pDC responses to PolyU are also downregulated *in vivo* but not *in vitro*

**Figure 5:**
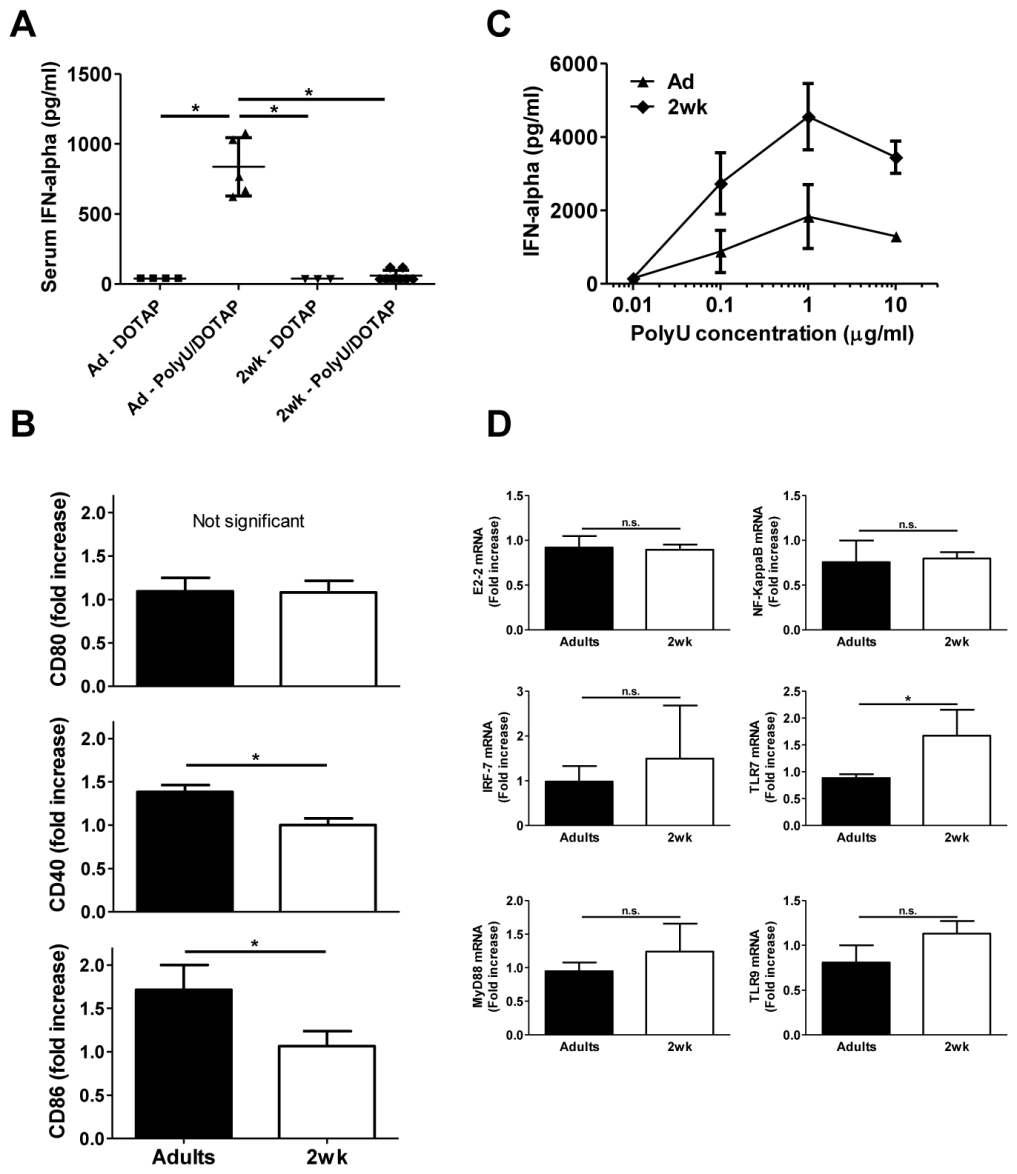
PolyU-stimulated function of infant pDCs is limited *in* *vivo* but not *in* *vitro*. (A) IFN-α titers quantified by ELISA in the serum 3h after i.v. injection of PolyU/DOTAP or DOTAP alone. One of two representative experiments is shown. *, *p*<0.05. (B) Fold increase of CD80, CD40 and CD86 expression by infant and adult splenic pDCs, determined by FACS staining between 0 and 3h after PolyU/DOTAP injection*, *p*<0.05. n = 5 to 8 mice per group. One of two representative experiments is shown. (C) Isolated untouched pDCs from infant or adult spleens were stimulated overnight with different concentrations of PolyU conjugated to polyethylenimine. Concentrations of IFN-α were tested in the supernatant by ELISA. n = 4 pools of 2 adult mice or 4 pools of 6 to 8 infant mice. One of two representative experiments is shown. (D) Expression of E2-2, IRF-7, MyD88, NF-κB, TLR7 and TLR9 mRNAs was analyzed by real-time RT-PCR in unstimulated and PolyU-stimulated (10μg/ml) splenic infant or adult pDCs. The results represent the fold increase derived from 3 independent experiments. n = 3 pools of 5 adult mice or 2 pools of 8 to 12 infant mice.

To define whether the limited infant pDC function was restricted to LCMV, an alternative *in vivo* model was developed using another TLR7 ligand: ssRNA (PolyU) encapsulated in DOTAP liposomes. PolyU effectively activated adult pDCs and induced high levels of serum IFN-α as previously described [36]. However, neither IFN-α production nor pDC activation was detected in infant mice (Figure 5 A and B) showing that infant pDC limitations are not specific to LCMV. The observation of a profound limitation of pDC activation, expansion and function in early life was in striking contradiction to the current consensus that neonatal murine pDCs have an adult-like capacity of producing IFN-I [37]. This common notion is based on normal pDC *in vitro* responses to PolyI:C [21], CpG-ODN or HSV-1 [22,23]. This was confirmed here, as PolyU *in vitro* stimulated infant pDCs expressed even higher IFN-α levels than adult pDCs (Figure 5 C) and at least adult levels of E2-2, IRF-7, MyD88, NF-κB, TLR7 and TLR9 mRNAs (Figure 5 D). Adult-like infant pDC responses were also elicited *in vitro* by imiquimod (TLR7 ligand, infant pDCs: 150 ± 106 pg/ml of IFN-α; adult pDCs: 139 ± 91 pg/ml) and CpG-A (TLR9 ligand, infant pDCs: 997 ± 6 pg/ml of IFN-α; adult pDCs: 750 ± 42 pg/ml). These discrepancies between adult-like *in vitro* and limited *in vivo* infant pDC responses raise the possibility that specific regulatory networks may be at play in the infant microenvironment. 

### IFN-α supplementation restores LCMV-specific T cell responses and viral control

**Figure 6:**
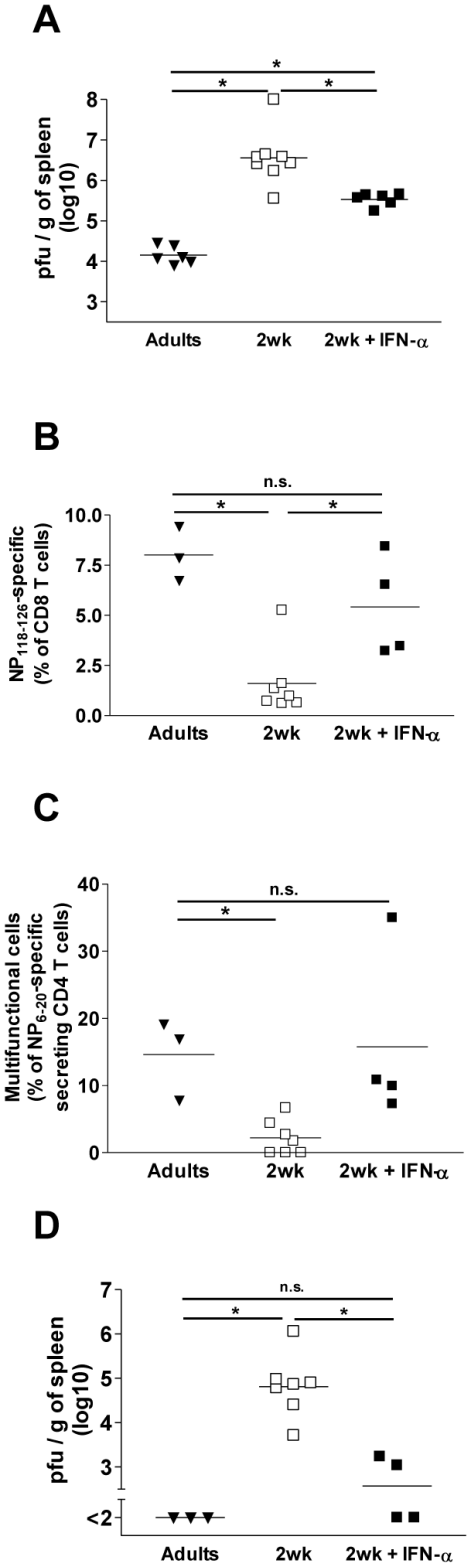
Restoration of viral control and LCMV-specific CD8^+^ and CD4^+^ T cell responses by IFN-α supplementation. (A) Spleen virus titer quantified 2 days after LCMV-WE infection of 2-week-old mice supplemented or not with 10,000U of recombinant mouse IFN-α given i.p. at 4h, 8h and 12h after infection. One of two representative experiments is shown. *, *p*<0.05. (B-D) One injection of long-lasting human recombinant pegylated IFN-α 2a was added 6h after LCMV infection. Analyses were performed 12 days later. (B) % of spleen LCMVnp118-126-specific CD8^+^ T cells. One of two representative experiments is shown. *, *p*<0.05. (C) % of LCMVnp6-20-specific IFN-γ, IL-2 and TNF-α secreting CD44^+^CD4^+^ T cells. Percentages obtained in medium controls were subtracted. One of two representative experiments is shown. *, *p*<0.05. (D) Splenic virus titers. One of two representative experiments is shown. *, *p*<0.05.

The need to avoid harmful inflammatory, alloreactive and autoimmune responses at a vulnerable period of life may explain the existence of distinct immune regulatory networks in early life. However, such benefits may be offset by the failure to overcome microbial challenge in early life. To obtain direct evidence of the critical contribution of IFN-I for early life infection control, we asked whether supplementing recombinant IFN-α early after infection would restore viral control. In a first series of experiments, 10’000U of recombinant murine IFN-α, was injected 4h, 8h and 12h after infection. Despite the short half-life (2.1 hours) of murine IFN-α this significantly reduced splenic LCMV-WE titers on day 2 (Figure 6A). To prolong the availability of recombinant IFN-α and assess its influence on T cell responses and viral control, we next added 1μg of long-acting (estimated half-life of 15h) pegylated human IFN-α 2a [38] at 6h post-infection. IFN-α supplementation significantly increased the frequency of LCMVnp118-126-specific CD8^+^ T cells (Figure 6 B) and of IFN-γ-producing LCMV-specific CD8^+^ T cells (data not shown), which approached or reached adult frequencies. Multifunctional CD4^+^ T cell responses were also restored to adult frequencies in IFN-α-treated 2-week-old infected mice (Figure 6 C). Accordingly, day 12 LCMV titers were significantly lower in IFN-α-supplemented infant mice (Figure 6 D). Thus, supplementing IFN-α early after LCMV-WE infection significantly improved early (day 2) and late (day 12) viral control and restored CD4^+^ and CD8^+^ early life T cell responses. This confirms the critical role of the lack of immediate-early IFN-α burst in the shaping of CD8^+^ and CD4^+^ T cell responses and in the protracted fate of LCMV-WE infection in early life.

## Discussion

In this study, we demonstrate that the vulnerability of infant mice to an acute pathogen such as LCMV-WE results from a limited immediate-early burst of IFN-I, which precludes early viral control and permanently impairs CD4^+^ and CD8^+^ T cell responses. These observations are novel and unexpected given the current understanding that pDC activation / IFN-I s are dispensable in adult hosts against acute viral pathogens such as LCMV-WE [15,28] and that neonatal murine pDCs have an adult response capacity when assessed *in vitro* [21-23]. The observation of an age-specific down-regulation of multiple critical pDC factors orchestrated by the E2-2 pDC regulator in response to LCMV or to model TLR7 agonists suggests that early life pDC responses are tightly regulated *in vivo*, possibly to avoid potentially harmful inflammatory or autoimmune reactions [6,7]. Remarkably, IFN-α supplementation restored T cell responses and virus control in infant mice.

IFN-I exert pleiotropic roles after pathogen recognition [10,39,40] such as blocking viral replication, infected cell apoptosis, NK and cDC activation, thus rapidly controlling viral loads [10-13,41]. In addition, IFN-I bridge innate and adaptive responses through a variety of mechanisms including the secretion of chemokines, enhanced Ag presentation, costimulation [42,43] and cross-priming [44,45]. They promote the expansion of CD4^+^ T cells [14,15] and may substitute for CD4^+^ T cell help [17,18]. These T cell responses have all been demonstrated as critical to the fate of adult LCMV infection, whereas their absence in the neonatal period (

< 48h) was shown 50 years ago as promoting immune tolerance and resulting into chronic infection [46]. We show here that the ontogeny of IFN-I responses affects the outcome of viral infections long after the neonatal period and that young age at infection may profoundly alter the host-pathogen balance

. 

Several lines of evidence suggest that the fate of early life LCMV infection is primarily controlled by the patterns of immediate-early IFN-I responses. First, LCMV viral titers were ≥ 1 log_10_ higher in infant than adult mice on day 2-3, a time at which viral control is controlled by innate immunity. At this early time point, splenic NK and cDC activation patterns were already adult-like in LCMV-infected infant mice, as reflected by similar IFN-γ serum titers and adult levels of IL-12 and IL-15 expression in infant cDCs (data not shown) despite limited IFN-I. This is in accordance with a previous study [47] suggesting that cDCs are only partially dependent on IFN-I for their maturation.

Next, LCMV-WE ran a similar course in 2-week-old BALB/c as in adult and infant IFNAR^-/-^ mice, confirming the importance of IFN-α signaling. Last, IFN-α supplementation supported early viral control, in accordance with its impact on HSV-1 in neonates [23,48]. Remarkably, IFN-α supplementation also restored the induction/expansion of adult-like CD4^+^/CD8^+^ responses, suggesting that its influence was not only mediated by a direct antiviral effect. Indeed, IFN-α injection restores CD8^+^ T cell responses in IRF7^-/-^ mice in which the early high viral load is a critical factor impairing CD8^+^ T cell responses to acute LCMV [49]. All these findings suggest that the limited immediate-early production of IFN-I by infant mice is insufficient to control early viral replication. Remarkably, the viral and immunological impacts of a transient delay in IFN-α release in infant mice were as severe as those resulting from the total absence of IFN-α signaling (Figure 2 and [50]). This may reflect the subsequent influence of higher levels of LCMV NP, which inhibit IFN-I production by infected cells [51] and thus terminate IFN-I responses, as serum IFN-I titers declined faster in infant mice. Thus, IFN-α is required before the peak of viral replication, a pattern successfully elicited by adult but not by infant LCMV-WE infected mice. 

Almost each virally infected cell may produce IFN-I in response to dsRNA, DNA, or viral nucleocapsids. However, pDCs are the earliest source of IFN-I in many viral infections [27,52-54]. pDCs were identified as the only source of the Day 1 peak of IFN-α during adult LCMV-Armstrong infection [25], DCs and infected cells contributing to Day 2-3 levels [30]. The failure of 2-week-old mice to generate early adult-like IFN-I responses to LCMV-WE reflects the specific pattern of their pDC responses. First, IFN-α titers were low on Day 1 but reached adult levels on Day 2, before declining on Day 3, probably under the inhibitory influence of LCMV NP [51]. Next, pDCs were rapidly activated by LCMV-WE infection in adult but not in 2-week-old mice, in which activation markers were undetectable 24h after infection- in accordance with the age-associated IFN-α response patterns. Thus, the delayed onset of IFN-I in LCMV-infected 2-week-old mice reflects the limited activation of their pDCs.

What may affect the *in vivo* IFN-I responses of infant pDCs? Their bone marrow development is not delayed and neonatal splenic pDCs are even more abundant than cDCs [21,22,37]. Flt3L and IFN-α supplementation increase their number, indicating the integrity of this developmental axis [23,48,55]. Thus, the postnatal maturation of pDCs is controlled by age-related regulatory mechanisms downstream of their BM production. The uniquely rapid IFN α/β response of adult pDCs has been ascribed to their capacity to retain TLR ligands in their endosome, to detect RNA and DNA viruses through the TLR7 and TLR9 endosomal sensors, i.e. without being infected [53], and to express high basal levels of IRF-7, the master regulator of MyD88-mediated IFN-I responses (review in [56]). All these critical molecules were downregulated in infant murine pDCs, both constitutively and during the course of LCMV-WE infection. This dampened early life pDC activation pattern correlated with an E2-2-orchestrated process limiting both IFN-I and NF-κB-mediated IL-6 and TNF-α responses. To our knowledge, this is the first demonstration that the expression of the E2-2 pDC regulator [34,35,57] may be modulated by a physiological process (age) – and the factors controlling the expression of E2-2 are yet unknown. The formal demonstration of a causal role for E2-2 would require forcing its expression in early life pDCs. However, the limited expression of E2-2 by infant pDCs appears as an attractive hypothesis for the observed down-regulation of TLR7/9, IRF-7, IRF-8 and IFN-I. Whether E2-2 also regulates the nuclear translocation of IRF-7 is unknown, and additional regulatory factors may contribute to the regulation of infant pDC activation. The TLR7 pathway was recently demonstrated as critical for the immediate-early production of IFN-I by pDCs [31]. We show here that a “natural” TLR7 ligand (PolyU) was similarly unable to elicit potent early life pDC responses *in vivo*. As neonatal murine pDCs do respond with adult-like IFN-I titers when exposed *in vitro* to TLR7/8 ligands such as Imiquimod or PolyU, immunoregulatory networks likely limit their activation *in vivo*. Neonatal B cells can produce high levels of IL-10 in response to TLR9 ligation, countering the production of proinflammatory cytokines by neonatal pDCs [58]. However, IL-10 was not secreted by infant B cells harvested up to 20h after LCMV-WE infection (data not shown), suggesting immunoregulatory influences independent of IL-10 producing B cells. Further studies should thus focus on the influence of the microenvironment on pDC activation to possibly identify yet undefined immune regulatory pathways in early life.

In adult hosts, the contribution of pDCs to the early innate control of viral infections varies: pDCs are critical against rapidly replicating viruses such as HSV [59], respiratory syncytial virus [60], mouse cytomeglovirus or vesicular stomatitis virus (VSV) [61] and rapidly growing LCMV strains such as Docile, whereas pDC-depleted E2-2^-/-^ mice rapidly clear slower replicating viruses such as LCMV-Armstrong [15]. In contrast, pDC activation appears critical for the fate of most early life viral pathogens: infant mice are highly vulnerable / fail to raise CD8^+^ T cell responses against viruses such as LCMV [9], VSV [62], influenza [63], HSV-1 [64] or chikungunya [65]. Thus, the immediate-early pDC activation / IFN-I burst appears much more critical for T cell activation and viral control in early than adult life. 

Numerous studies have reported the reduced *in vitro* responses of human cord blood pDCs to TLR7 or TLR9 ligands [66-70], suggesting the existence of intrinsic pDC limitations persisting until at least 6-12 months of age [68]. Functionally, an inverse correlation was reported between circulating pDC numbers and the clinical severity of lower respiratory tract infections in infants and young children [71]. Interestingly, a recent study noted that the blunted *in vitro* responses of cord blood pDCs had been observed with whole blood or enriched (

< 50% pure) pDC populations and showed that >99% pure cord blood pDCs do raise adult-like IFN-α responses to TLR7 or TLR9 stimuli [72]. Thus, neonatal human pDCs are intrinsically able to generate adult-like type-I IFN responses in vitro but are influenced by exogenous factors, as observed in mice. Our observations extend on previous findings by demonstrating that such immunoregulatory factors may be sufficiently potent in vivo to impair viral control and T cell immunity. As pDCs are involved in inflammatory responses which may be detrimental to the neonatal brain and IFN-I contributes to increasing the risks of autoimmunity [73], it is biologically plausible that the slow onset of pDC activation and IFN-I responses has beneficial effects. The downregulation of early life in vivo pDC responses to TLR7 ligands identified in this report may thus have been evolutionary driven by immunological safety considerations. Although we show here that novel strategies to supplement or stimulate immediate-early IFN-α responses, i.e. through adjuvantation, may exert potent protective effects through a sustained impact on CD4^+^ and CD8^+^ T cell responses, this will thus require a careful d

emonstration of their immune safety. 
